# Executive Function Deficits and Social-Behavioral Abnormality in Mice Exposed to a Low Dose of Dioxin *In Utero* and via Lactation

**DOI:** 10.1371/journal.pone.0050741

**Published:** 2012-12-12

**Authors:** Toshihiro Endo, Masaki Kakeyama, Yukari Uemura, Asahi Haijima, Hiroyuki Okuno, Haruhiko Bito, Chiharu Tohyama

**Affiliations:** 1 Laboratory of Environmental Health Sciences, Center for Disease Biology and Integrative Medicine, Graduate School of Medicine, The University of Tokyo, Tokyo, Japan; 2 Department of Biostatistics, School of Public Health, The University of Tokyo, Tokyo, Japan; 3 Department of Neurochemistry, Graduate School of Medicine, The University of Tokyo, Tokyo, Japan; Roma Tre University, Italy

## Abstract

An increasing prevalence of mental health problems has been partly ascribed to abnormal brain development that is induced upon exposure to environmental chemicals. However, it has been extremely difficult to detect and assess such causality particularly at low exposure levels. To address this question, we here investigated higher brain function in mice exposed to dioxin *in utero* and via lactation by using our recently developed automated behavioral flexibility test and immunohistochemistry of neuronal activation markers Arc, at the 14 brain areas. Pregnant C57BL/6 mice were given orally a low dose of 2,3,7,8-tetrachlorodibenzo-*p*-dioxin (TCDD) at a dose of either 0, 0.6 or 3.0 µg/kg on gestation day 12.5. When the pups reached adulthood, they were group-housed in IntelliCage to assess their behavior. As a result, the offspring born to dams exposed to 0.6 µg TCDD/kg were shown to have behavioral inflexibility, compulsive repetitive behavior, and dramatically lowered competitive dominance. In these mice, immunohistochemistry of Arc exhibited the signs of hypoactivation of the medial prefrontal cortex (mPFC) and hyperactivation of the amygdala. Intriguingly, mice exposed to 3.0 µg/kg were hardly affected in both the behavioral and neuronal activation indices, indicating that the robust, non-monotonic dose-response relationship. In conclusion, this study showed for the first time that perinatal exposure to a low dose of TCDD in mice develops executive function deficits and social behavioral abnormality accompanied with the signs of imbalanced mPFC-amygdala activation.

## Introduction

The development of the brain is highly sensitive to be perturbed by various kinds of environmental factors including chemical exposure [1], [2], [3], [4]. These environmental factors may account for an increasing prevalence of children's mental problems such as learning disability [5], autism spectrum disorders (ASD) [6], and attention-deficit/hyperactivity disorder [7]. However, there is a scarcity of direct evidence to link brain dysfunctions with clinically measurable mental and behavioral abnormalities induced by chemical exposure at an environmentally-relevant level.

Among the environmental chemicals, dioxin has long been a potential threat to the brain function and behavior of children [8], [9]. Dioxins are a group of chemicals unintentionally produced in combustion processes or byproducts of manufacturing certain kinds of herbicides. Due to their persistency in the environment, humans have been exposed to non-negligible amounts of dioxins mainly through daily food [10]. Clinical and epidemiological studies have revealed the occurrence of a variety of signs and symptoms in dioxin-exposed human populations, including disturbances in psychomotor and neurobehavioral functions in children [8], [9], [11]. *In utero* and lactational exposure of rodents to 2,3,7,8-tetrachlorodibenzo-*p*-dioxin (TCDD), the most toxic congener among dioxin family, induces abnormal sexually dimorphic behaviors in adulthood [12], [13], [14], [15], [16], [17], and alters learning behaviors [18], [19], [20], [21], [22], [23], [24], [25], [26]. Previous studies have shown that the cerebral cortex is a vulnerable target to TCDD insult. For example, the expression of NMDA receptor subunits in the neocortex and hippocampus, and activity-dependent expression of brain derived neurotrophic factor in the neocortex was altered in rats exposed to TCDD *in utero* and via lactation [15], [27]. Recently, Mitsuhashi and associates [28] showed that *in utero* exposure of mice to TCDD impairs histogenesis of the neocortex, albeit at a high dose corresponding to approximately one-ninth of its 50% lethal dose (LD 50) [29]. However, it is largely unknown whether and how exposure to dioxin, particularly at a low dose, affects higher brain function.

As higher brain function, executive function and social brain function require a particular attention because impairment of these functions has been implicated in a variety of neurodevelopmental disorders and psychiatric illnesses [30], [31], [32], [33], [34], [35], [36]. Executive function is a set of cognitive processes responsible for organizing appropriate goal-directed actions in an ever-changing environment, which is subserved by the prefrontal cortex [36], [37], [38], [39], [40]. To evaluate executive function in mice, we have recently established the behavioral flexibility test using IntelliCage, a fully automated behavioral testing apparatus for mice under a group-housed condition [41]. This test provides comprehensive and reproducible indices of behavioral flexibility over a variety of time scales (i.e., intervals of hours, days, and weeks). Brain function responsible for social behavior is also one of the important roles of the cerebral cortex [42], [43], [44], [45]. In the present study using IntelliCage, competitive dominance for limited sources of water reward after water deprivation was also analyzed to investigate the possible alterations in social status in a group-housed environment. It is important to elucidate the biological basis that determines individual's social status because social status is associated with a wide variety of stress-related health problems, such as hormonal, reproductive, immunological, and cognitive dysfunctions [46]. The brain areas associated with cognition of ‘social hierarchy’ were found in the dorsolateral and medial prefrontal cortex in humans [45]. In addition, Wang and associates [47] showed that ‘social hierarchy’ in mice can be controlled by manipulating the synaptic efficacy in the medial prefrontal cortex (mPFC) neurons. Thus, executive function and competitive dominance are worth investigating as possible targets of chemical exposure which is suspected to affect the development of higher brain function.

In this study, we found that *in utero* and lactational exposure to a low dose of TCDD, which corresponds to approximately 1/300^th^ of the LD50 and is comparable to the dose from which was used to derive a tolerable daily intake level in humans [48], perturbs executive functions and induce low competitive dominance for limited sources of water reward after water deprivation of offspring, and that such behavioral alterations are consistent with altered signs of neuronal activity in the mPFC and amygdala.

## Materials and Methods

### Reagents and Chemicals

TCDD (purity >99.5%) was purchased from Cambridge Isotope Laboratory (Andover, MA, USA). Antibodies for c-Fos and NeuroTrace were obtained from Santa Cruz Biotechnology (Santa Cruz, CA, USA) and Invitrogen Japan (Tokyo, Japan), respectively. A rabbit polyclonal anti-Arc antibody (OP-1, crude) was produced as described previously, and the specificity of the anti-Arc antibody was verified by Western blotting (Fig. S1) and immunocytochemistry [49]. Biotinylated anti-rabbit IgG was obtained from Vector Laboratories (Burlingame, CA, USA). Reagents for tyramide signal amplification were obtained from Perkin Elmer (Waltham, MA, USA). The other reagents were obtained from Nakalai Tesque (Kyoto, Japan).

### Animals and Treatment

Pregnant C57BL/6 mice purchased from CLEA Japan (Tokyo, Japan) were housed in the animal facility with temperature at 22–24°C and humidity at 40–60%, on a 12/12 hr light-dark cycle (lights on: 8:00–20:00). Laboratory rodent chow (Lab MR Stock, Nosan, Yokohama, Japan) and distilled water were provided *ad libitum* unless otherwise specified. In the behavioral test using IntelliCage, the access to water, not diet, was restricted and permitted during the 3-hr test period (22:00–1:00) per day only. The pregnant mice were orally administered vehicle (corn oil containing 0.6% n-nonane) or TCDD in vehicle at a dose of 0, 0.6 or 3.0 µg/kg once on gestation day 12.5. This mode of exposure causes *in utero* and lactational exposure of offspring organs including brain [18], [50]. The pups were culled to eight per dam at postnatal day (PND) 0, weaned at PND 21, and kept under the same conditions thereafter as their dams. A male offspring was randomly selected per dam for the behavioral flexibility test using IntelliCage to minimize litter effects. Three groups, named Control, TC-0.6, and TC-3.0, were prepared, according to the doses given to their dams (n = 8/group). At the postnatal day 180, they were lightly anesthetized with diethyl ether and implanted subcutaneously a glass-covered transponder having a unique ID code (Datamars, SA, USA) for radiation frequency identification (RFID) for behavioral experiments using IntelliCage. For the competition task using IntelliCage, open field test and rota rod test, and, a male offspring, which was a littermate used in the behavioral flexibility test, was randomly selected per dam to make another set of three groups (Control, TC-0.6, and TC-3.0; n = 8/group). The experimental protocols of animal experiments of this study were approved by the Animal Care and Use Committee of the Graduate School of Medicine of the University of Tokyo.

### IntelliCage Apparatus

**Figure 1:**
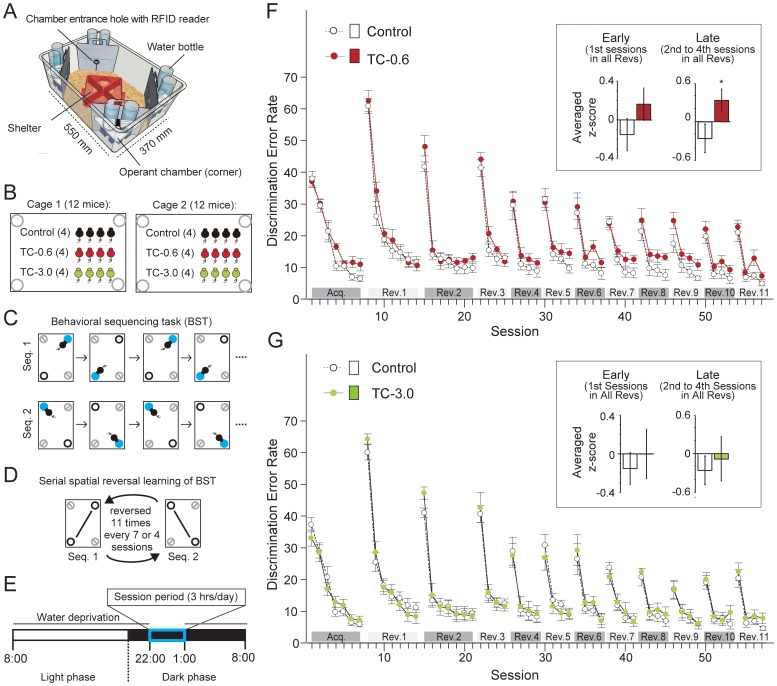
Impaired behavioral flexibility in TCDD-exposed mice (inter-session analysis). (A) Overview of IntelliCage apparatus. (B) Group composition of mice housed and tested in each IntelliCage apparatus. (C) Behavioral sequencing task. Mice were allowed to obtain water reward for 4 seconds when they visited an “active” rewarded corner (blue circle). The location of the active rewarded corner was alternately switched between the two diagonally positioned corners each time the mouse received a reward. Thus, the mice had to acquire the behavioral sequence of alternating between the two rewarded corners to continuously obtain rewards. A visit to the never-rewarded corners (gray circles with a diagonal line) was counted as a discrimination error. (D) Serial reversal task. For each mouse, the assigned spatial patterns of the rewarded corners (seq. 1 or seq. 2) were reversed 11 times every 7 or 4 sessions. (E) Time-line of the experiment for each day. (F, G) Learning performance on the behavioral flexibility test. For the purpose of readability, data from the TC-0.6 and TC-3.0 groups of mice were separately plotted in F and G, respectively, whereas the data from the Control group are shown in F and G. Discrimination error rates (the number of discrimination errors in the first 100 corner visits in the session) are indicated as the means ± S.E.M. (n = 8/group). For each session, the individual mouse's discrimination error rate was transformed into a z-score calculated among all the mice. The bars in the insets in F and G indicate the averaged z-scores for each group in the first sessions of all the Revs (early stage of reversal learning) and in the second to fourth sessions of all Revs (late stage of reversal learning). * indicates a significant difference from the Control group (*P*<0.05, ANOVA followed by Tukey's post hoc test).

IntelliCage (TSE Systems GmbH, Bad Homburg, Germany) is a computer-based, fully automated testing apparatus that can be used to monitor the spontaneous and cognitive behavior of group-housed RFID-tagged mice housed in a large home cage (Fig. 1A and S2A) [51]. In short, a large plastic cage (55×37.5×20.5 cm^3^) is equipped with 4 triangular operant chambers (corners, hereafter) (15×15×21 cm^3^). RFID readers, infrared sensors, and lickometers has a capability to perform simultaneous monitoring of as many as 16 RFID-tagged mice in an IntelliCage apparatus. In this unit, only one mouse can enter a corner at a time because of the size of the tunnel and corner. In the inner space of the corner, mice can have access to two nose-poke holes equipped with an infrared beam-break response detector. A nose poke at the hole triggers the opening of a motorized access gate to water-bottle nipples (gate, hereafter) (Fig. S2B). In IntelliCage, the time and duration of each behavioral event (corner visit, nose poke, and licking), mouse ID and corner ID were automatically recorded through RFID readers, infrared sensors and lickometers.

### IntelliCage Test Procedures

#### Acclimation

All the groups of mice were introduced to IntelliCage apparatuses at 10:00 a.m. on the same day. In each IntelliCage apparatus, the numbers of mice were counterbalanced among groups (see Fig. 1B for the behavioral flexibility test and 4G for the competition task). Then, the acclimation and behavioral tests were conducted by the following procedures. During the acclimation phase 1 (3 days), the gates through which mice can have access to water-bottle nipples in all the corners were kept opened, and thus, the mice were allowed to have water in each corner *ad libitum*. In this phase, indices of exploratory and spontaneous activities and the circadian index [52] were analyzed (Table S1). In the acclimation phase 2 (1 day), the mice were trained nose poking behavior. In this phase, all the gates in front of the water bottles were closed initially so that mice had to nose poke to open the gate and drink water. The gate could be opened only by the first nose poke per corner visit, and was closed 4 seconds later. Mice could obtain water by a nose poke throughout the day. In the acclimation phase 3 (5 days), mice were given a chance to open the gate by a nose poke during a 3-hr period (22:00 to 1:00) per day. Beside default LEDs in each corner, we put an additional blue LED on a side wall of the cage to let mice know the session period: the blue LED was turned on throughout the 3-hr period. Likewise, during this period, red LEDs in the corner were turned on when a mouse made a visit, and turned off when the gate was closed. During acclimation, all the mice visited all the four corners extensively. Because their preferences to a particular corner among four corners varied between 20–30% (chance level = 25%), all the mice were considered to get accustomed evenly to all the corners (Table S1).

#### Behavioral Flexibility Test

The experimental procedure was essentially the same as the one used in the previous study [41]. Briefly, the behavioral flexibility test was composed of the acquisition phase and its serial reversal task phase. The former and the latter consist of behavioral sequencing task (Session 1–7, Fig. 1C) and repetitions of reversal task (Rev. 1–Rev. 11, Fig. 1D), respectively. The water-deprived mice had 4-second access to water as a reward when they visited the assigned corners during the 3-hr test session (22:00–1:00) per day (Fig. 1E). A total of 57 sessions were conducted. In each session, mice could gain rewards continuously by alternating the visits between the two diagonally positioned rewarded corners (Fig. 1C). Each of the two rewarded corners had two distinct states, “active” or “inactive”, in a mutually exclusive manner. That is, for a given mouse, there is always one “active” rewarded corner, one “inactive” rewarded corner, and two never-rewarded corners at the same time. A mouse was allowed to open a gate in an “active” rewarded corner by a nose poke and drink water for 4 seconds. After that, the corner became “inactive” instantaneously, and this signal was synchronized to change the other rewarded corner, which was previously “inactive”, to become “active”. The alternation of the corner assignment was controlled for each mouse independently by the IntelliCage software program. Thus, the mice had to shuttle between the two diagonally positioned rewarded corners to acquire rewards continuously. A visit to the never-rewarded corner was regarded as a discrimination error. The number of discrimination errors within the first 100 visits in each session ( = discrimination error rate) was utilized for inter-session comparisons of learning performance. All the corner assignments were counterbalanced among groups of mice in each cage so that no specific corners would receive more traffic than others. For the evaluation of the behavioral abnormality of useless repetitive nose poking, we extracted the number of nose pokes per visit to either the rewarded corner or non-rewarded corner, i.e., neutral and never-rewarded corner, within the first 100 visits by utilizing filter function of the IntelliCage Analyzer software (TSE Systems GmbH, Bad Homburg, Germany). For each session, the number of nose pokes of each mouse that entered rewarded corner or non-rewarded corner was averaged on the group basis. The excessive number of nose pokes per visit, i.e., more than two nose pokes per visit, to the rewarded and non-rewarded corner was defined as compulsive and impulsive repetitive nose poking behavior, respectively. To evaluate competitive dominance for the limited sources of water reward, corner visit frequencies per minute were calculated by Excel (Microsoft) from ‘visit file’ exported from the IntelliCage Analyzer software.

#### Competition Task

This task differed from that of the behavioral flexibility test in that mice could drink water at any of the corners during the 3-hr period. To investigate the effects of variation in social environment on the competitive dominance index, all of the mice were tested under a highly competitive condition on Days 1–4 (14 mice per cage), under a less competitive condition on Days 5–8 (7 mice per cage), and again under a highly competitive condition (14 mice per cage) on Days 9–12. Acclimation of mice to IntelliCage apparatus was conducted in the same way as the behavioral flexibility test. After acclimation, the task lasted for 12 days. In the task, mice were deprived of water for 21 hr per day, and allowed to drink water for 4 seconds per corner visit during 22:00–1:00. In the same way as the behavioral flexibility test, an additional blue LED was fixed on a side wall of the cage and used as a signal during the 3-hr period. The number of corner visits during the first five minutes in each session was used as an index to assess the level of competitive dominance of mice in the competition for water reward.

### Open Field Test

A dimly lighted (15 lux), open field (50×50×50 cm^3^) was used to assess the basal activity level in a novel environment for 60 min. The “center area” was defined as 25 cm×25 cm area located at the center of the open field (Fig. S3).

### Rota Rod Test

A rota rod treadmill machine MK-670 (Muromachi Kikai Co., Tokyo, Japan) and a 30-mm-diameter rotating bar for mice were used to assess motor coordination. First, a consecutive three-day training was conducted followed by a consecutive four-day session per mouse. In the training, each mouse was put on a rotating bar 4 times a day. The speed of the rotating bar was linearly increased in each 60-second trial from 3 rpm at the start to 3, 10, 20, and 20 rpm at the end of Trial 1, 2, 3, and 4, respectively. In the test, each mouse was put on a rotating bar 4 times a day, the speed of which was linearly increased in each 60-second trial from 3 rpm at the start to 10, 20, 30, and 40 rpm at the end of Trial 1, 2, 3 and 4, respectively (Fig. S3).

### Immunohistochemistry

To analyze immunohistochemically the signs of neuronal activation in specific brain areas, mice were killed by cervical dislocation 1 hr after the last session started. Brains were collected and immediately frozen in powdered dry ice, and stored at −80°C. Frozen brain tissues were cryosectioned to make 20-µm thick sections at −20°C (Leica, Tokyo, Japan). Anti-Arc and anti-c-Fos antibodies were used to detect Arc and c-Fos as neuronal activation marker proteins, and fluorescent Nissl staining was performed using NeuroTrace to count neuronal cell numbers. The rest of the procedures were essentially the same as described in a previous study [53]. Briefly, brain sections on slides were washed in 0.05% Triton ×100 in phosphate-buffered saline (PBST), and then fixed in 4% paraformaldehyde for 10 min. The sections were incubated in a solution containing 0.3% H_2_O_2_ in methanol for 30 min to quench endogenous peroxidase activity. After incubation with a primary antibody (anti-c-Fos, 1∶1000; anti-Arc, 1∶10000), Tyramide signal amplification (TSA Biotin System, Perkin Elmer, Waltham, MA, USA) was then performed. Finally, the signals were visualized with 3,3′-diaminobenzidine (DAB). For fluorescent Nissl staining, the sections were incubated with NeuroTrace (1∶200) for 1 hr, followed by washing in PBS for 5 min, and mounted for fluorescence microscopy.

### Quantification of Immunolabeled Cells

Stereological analysis for immunolabeled cell counting was conducted utilizing a semiautomated optical fractionator method with Stereo Investigator software (Microbrightfield Williston, VT, USA). Cell counts were conducted in the following 14 areas: prelimbic cortex (PrL), orbital cortex (Orb), infralimbic cortex (IL), anterior cingulate cortex (ACC), dorsal lateral caudate putamen (dlCPu), nucleus accumbens core (AcbC), primary motor cortex (M1), primary somatosensory cortex barrel field (S1BF), retrosplenial granular cortex (RSGc), ectorhinal cortex (Ect), CA1 field of hippocampus (CA1), CA3 field of hippocampus (CA3), basolateral nucleus of the amygdala (BLA), and central nucleus of the amygdala (CeA). The boundary between areas was determined by referring to the mouse brain atlas [54] in each section at low magnification using Stereo Investigator software. Cells were counted in a semiautomated fashion under a 40-fold magnification. Cell densities in each brain area were then automatically estimated in 3D from three consecutive sections using the same software. The group average numbers of Arc- and c-Fos-positive cells in each area were calculated from five brains of mice per group.

### Statistical analysis

For both behavioral and immunohistochemical data analyses, one-way or two-way ANOVA followed by Tukey's HSD post hoc test for multiple comparisons was used. *P*-values at 0.05 or less were considered statistically significant. All the statistical analyses were carried out by using SAS (Cary, NC, USA) and SPSS (Chicago, IL, USA) software. To study the possible relationships among the behavioral parameters in this experiment, we made a correlation coefficient matrix for 16 behavioral variables which included indices of basal activity, water licking, behavioral flexibility, compulsive and impulsive repetitive behavior, and competitive dominance (for each description, see Table S2). Correlation coefficient was calculated using z-scores of each behavioral parameter (Fig. S4).

## Results

### Normal Basal Activity Levels and Motor Coordination

No dams or their offspring were found to develop significant abnormalities in general health parameters including the body weight gain of the dams during pregnancy, maternal death, the number of live pups, and the birth weight of the pups. In addition, no significant differences in the levels of exploratory or spontaneous activities and the motor coordination of the offspring were found among the three groups (Control, TC-0.6, and TC-3.0) in the acclimation phase of the behavioral flexibility test, open field test, and rota rod test (Fig. S3, Table S1). These observations are consistent with our previous study [18].

### Impaired Behavioral Flexibility in TCDD-exposed Mice

In the acquisition phase, the discrimination error rate of each of the three groups of mice was found to decrease to approximately 10% by Session 7, showing that they equally acquired the behavioral sequencing task (Figs. 1F and 1G). In the subsequent serial reversal task, the discrimination error rate of each group was found to be clearly elevated in the first session in each Rev., reflecting that each group of mice firmly acquired the behavioral sequence assigned to the mice in the previous phase.

To assess behavioral flexibility, we defined the first session and the 2nd to 4th sessions in each Rev. as the early and late stages of reversal learning, respectively. For each session, the individual mouse's discrimination error rate was transformed into a z-score calculated among the discrimination error rates of all the groups of mice in the session. For each group, the z-scores were averaged for all the early stage sessions (the first sessions of Rev. 1–Rev. 11) and for all the late stage sessions (the 2nd to 4th sessions of Rev. 1–Rev. 11). These averaged z-scores were used for comparison among groups (insets in Figs. 1F and 1G). It was found that the TC-0.6 group had a significantly higher z-score of error rate in the late stage than the Control group (Fig. 1F), showing that the TC-0.6 group attained a lesser degree of reversal leaning than the Control group in the late stage.

**Figure 2:**
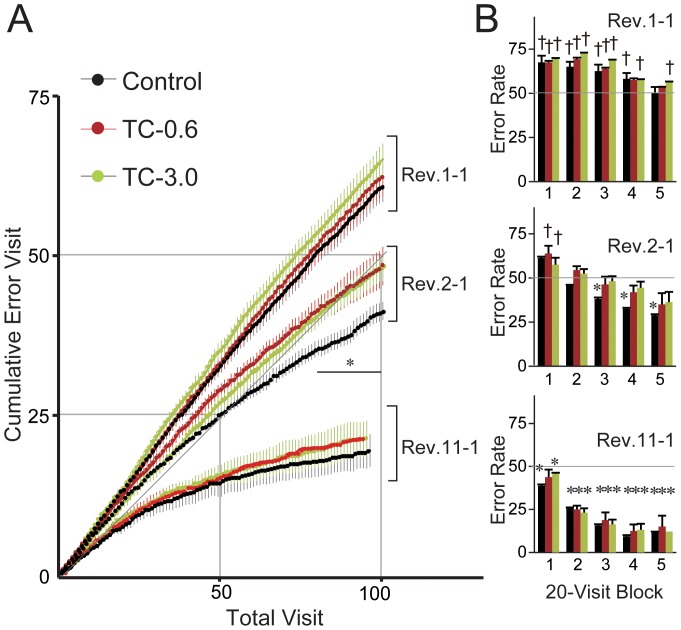
Impaired behavioral flexibility in TCDD-exposed mice (intra-session analysis). (A) The curves of cumulative error visit (mean ± S.E.M., n = 8/group) in the first sessions of Rev. 1, 2, and 11 (Rev.1-1, Rev.2-1, and Rev.11-1, respectively) of each group are shown. * represents a significantly lower cumulative error visit than chance in the Control group within a range of 80th–100th total visit (*P*<0.05, repeated ANOVA). (B) Error rates per 20-visit block in Rev. 1-1, Rev. 2-1, and Rev. 11-1 (mean ± S.E.M., n = 8/group). The first 100 corner visits in each session were divided into five blocks (block 1 to 5) and analyzed using factorial ANOVA. The black, red and green bars indicate the data from the Control, TC-0.6, and TC-3.0 groups, respectively. † and * indicate a significantly higher or lower difference from chance (50%, gray line), respectively.

In the early stage of reversal learning, both TC-0.6 and TC-3.0 groups showed a tendency to have higher z-score than the Control group. Thus, we next performed detailed intra-session analyses of the early stage using the cumulative error visit and 20-visit block error rate, as indicators (Fig. 2). We selected the first sessions of Rev. 1, Rev. 2, and Rev. 11 in each group for intensive analysis for the following reasons. The first session of Rev. 1 (Rev. 1-1) is considered to be unique because mice were forced to shift their behavior to a new behavioral sequence for the first time. The first session of Rev. 2 (Rev. 2-1) has also a unique feature in that it is the session in which mice relearned the previously acquired behavioral sequence for the first time. During the subsequent Revs., mice were found to gradually make their behavioral shift to a new sequence more efficiently by repeating the reversal task. Thus, we selected to analyze the performance of the first session in the final Rev. (i.e., Rev. 11-1, in this study) as a representative of the over-trained learning phase. In Rev. 1-1, each of these three groups of mice made error visits at a higher frequency than chance throughout the session, indicating that these mice, on a group basis, strongly adhered to the behavioral sequence that they had acquired in the acquisition phase, and could not adapt their behavior to the reversal task. In Rev. 2-1, the increase rate of the cumulative error visit and the 20-visit block error rate of the three groups of mice were remarkably decreased compared with the corresponding groups in Rev. 1-1. Thus, these mice, on a group basis, were shown to adapt their behavior to the reversal task within a session of Rev. 2-1. However, the degree of behavioral adaptation in this session differed among the groups. In the Control group, the cumulative error visit was significantly decreased less than chance within the session (Fig. 2A), and the 20-visit block error rate was also significantly decreased than chance as early as block 3 (Fig. 2B). In contrast, both TC-0.6 and TC-3.0 groups could not improve their performance better than chance in these indices within the session. In Rev. 11-1, the final Rev., the three groups of mice were found to have attained a rapid behavioral shift to the reversed contingency. These results show that *in utero* and lactational exposure to TCDD induces a delay in the process of attaining rapid behavioral shift in the serial reversal task.

### Compulsive Repetitive Nose Poking in Mice Exposed to a Low TCDD Dose

**Figure 3:**
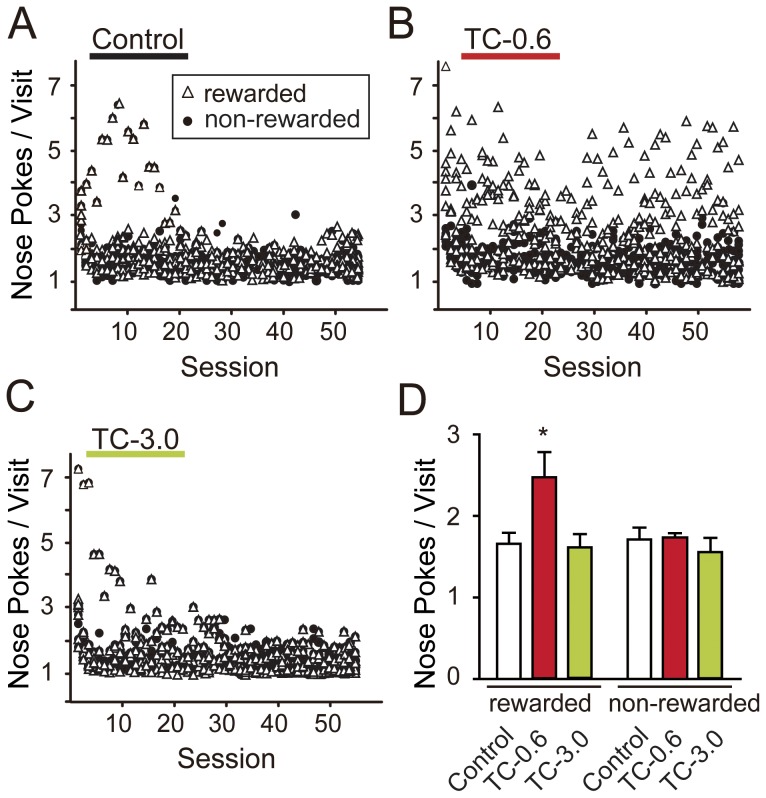
Compulsive repetitive nose poking in mice exposed to a low TCDD dose (TC-0.6). (A, B, C) The number of nose pokes per rewarded visit (open triangle) and non-rewarded visit (closed circle): (A) Control, (B) TC-0.6, (C) TC-3.0. Each plotted point indicates the average number of nose pokes per visit made by an individual mouse in a session. (D) Group-averaged numbers of nose pokes throughout the sessions per rewarded visit or non-rewarded visit. Error bars indicate ± S.E.M., n = 8/group. * indicates a significant difference from the Control and TC-3.0 groups (*P*<0.05, ANOVA, followed by Tukey's post hoc test).

In this test, nose pokes of more than twice per corner visit were considered useless behavior as they did not result in additional rewards. We defined the number of nose pokes per visit to rewarded and non-rewarded corner as indices of compulsive and impulsive repetitive behavior, respectively. The mice belonging to the Control and TC-3.0 groups learned not to make useless nose pokes, regardless of rewarded or non-rewarded visits, during the 57 sessions (Figs. 3A and 3C). In contrast, the TC-0.6 group mice made multiple useless nose pokes in the rewarded corner throughout the sessions (Fig. 3B). When the average number of nose pokes throughout the sessions was compared, the TC-0.6 group had a significantly greater number of nose pokes at rewarded visits than the Control and TC-3.0 groups (Fig. 3D). In contrast, such behavior was not observed in non-rewarded visits. Thus, the TC-0.6 group was shown to have compulsive repetitive nose poking responses as a sign of a deficit in executive function, while the TC-3.0 group did not.

### Low Competitive Dominance in Mice Exposed to a Low TCDD Dose

**Figure 4:**
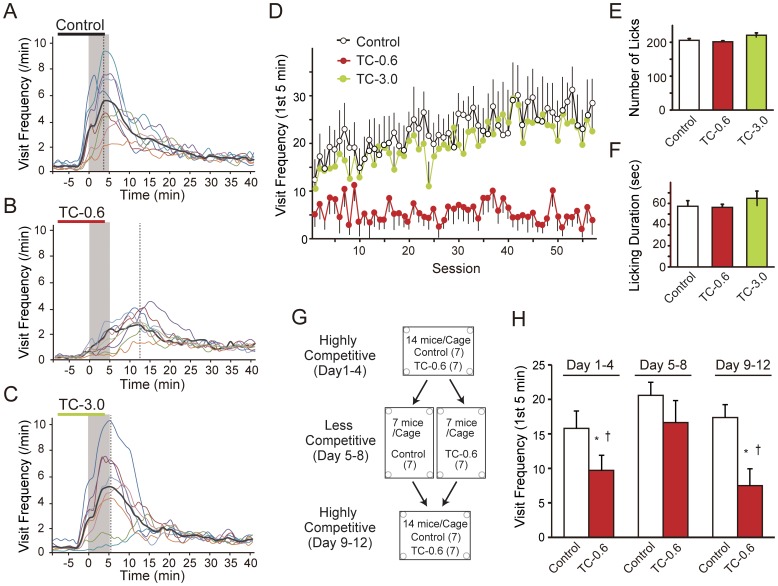
Low competitive dominance in the low TCDD dose (TC-0.6) group. (A, B, C) Time-course of visit frequency in the first session of the day (21:50–22:40). The gray-colored period indicates the first five minutes (22:00–22:05) of the task. Vertical dotted lines indicate a peak in the group-averaged number of visits. (A, B, C) Time-course of visit frequency at the beginning of the session (21:50–22:40). Colored lines indicate the averaged visit frequency across all the sessions of each mouse, and the black thick lines indicate the average of each group: (A) Control, (B) TC-0.6, and (C) TC-3.0. (D) Visit frequency in the first five minutes throughout the sessions for each group (mean ± S.E.M., n = 8/group). (E, F) Average number of visits and duration of licking per session are shown as indices of daily water consumption. Error bars indicate ± S.E.M., n = 8/group. (G) A diagram of the competition task, in which the Control and TC-0.6 groups of mice (littermates of the mice used in the behavioral flexibility task) were subjected to the same water deprivation schedule as in the behavioral flexibility task. From days 1 to 4, all of the mice (a total of 14 mice, comprised of the Control and TC-0.6 groups) were housed in the same IntelliCage apparatus (a highly competitive condition). From days 5 to 8, each group of mice was housed separately in two different IntelliCage apparatuses (a less competitive condition). From days 9 to 12, all the mice were again housed in the same IntelliCage apparatus (a highly competitive condition). (H) Visit frequency in the first five minutes in the competition task. * and † indicate a significant difference from the Control group in the identical time period and from the TC-0.6 group on days 5 to 8, respectively. Error bars indicate ± S.E.M., n = 7/group.

At the start of each session, the mice were observed to make intensive visits to the four corners of the apparatus competing against each other, presumably due to thirst after a water deprivation period. However, the mice in the TC-0.6 group made far less corner visits than the other two groups, especially during the first 5 minutes (Figs. 4A, 4B, and 4C). In addition, the number of corner visits made by the TC-0.6 group peaked approximately 5 minutes later than the other groups. The suppressed corner visit behavior during the first 5 minutes of the task of the TC-0.6 group was clearly demonstrated throughout the sessions in a robust, non-monotonic dose-dependent manner (Fig. 4D). As the total number of lickings and the duration of water licking in each session were not different among the groups, it was unlikely that the motivation of the TC-0.6 group for the water reward was affected by TCDD (Figs. 4E and 4F). Since the time spent per corner visit was not found to differ among the three groups during the first three 5-minute periods after cessation of deprivation of water (22:00–22:05, 22:05–22:10, 22:10–22:15), the significant reduction in visit frequency of the TC-0.6 group was not likely to be due to differences in the dwelling time at the corners (Fig. S5).

To confirm whether the suppressed competitive behavior in the TC-0.6 group was attributable to social factors under the group-housed condition, the competition task was conducted using the littermates of the mice used in the behavioral flexibility test (Fig. 4G). Again, the TC-0.6 group made a significantly lower number of corner visits than the Control group during the first 5 minutes after cessation of water deprivation (Fig. 4H, Days 1 to 4). However, the suppressed corner visit behavior was not observed when each group was separately housed (Days 5 to 8). When the Control and TC-0.6 groups of mice were housed together again, the TC-0.6 group showed a significantly lower number of corner visits than the Control group (Days 9 to 12). These results show that the suppressed corner visits of the TC-0.6 group were not due to their unawareness or apathy at the start of the session, but, instead, were dependent on social context, i.e., the presence of the other group of mice.

### Brain Areas Associated with TCDD-induced Behavioral Alterations

**Figure 5:**
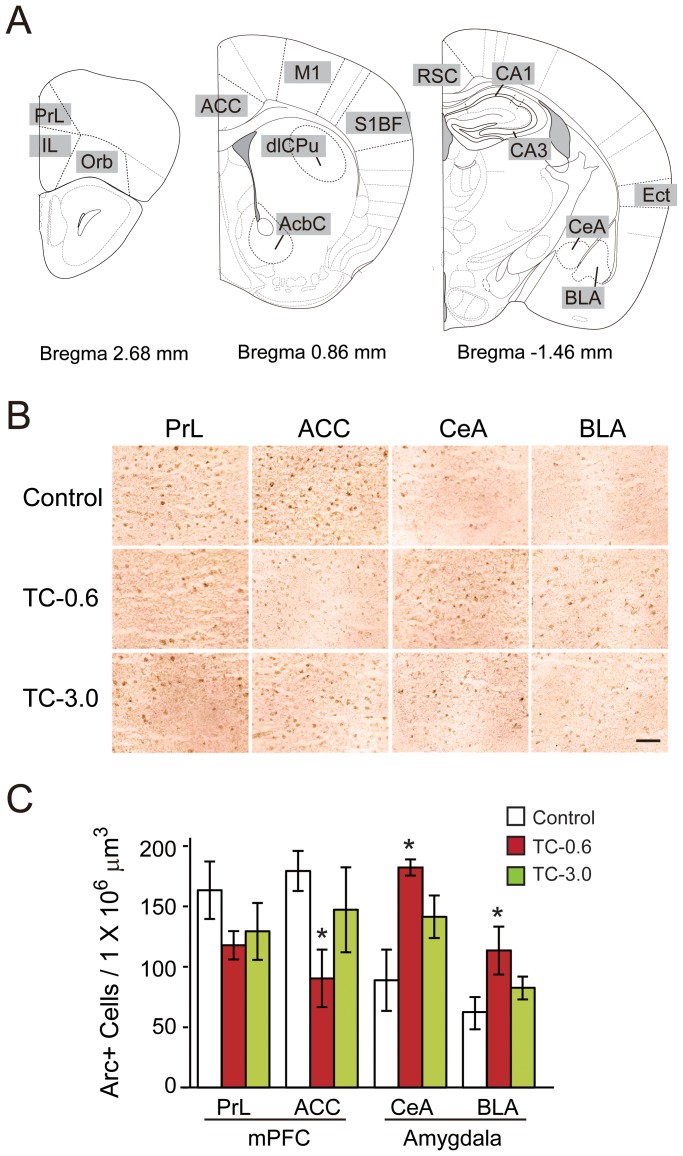
Brain areas associated with TCDD-induced behavioral alterations. (A) Regions of interest. Illustrations are modified with permission from [54]. (B) Representative photomicrographs of Arc-positive cells in the PrL, ACC, CeA, and BLA. (C) Numbers of Arc-positive cells in each brain area as estimated by stereological analysis. Bars, open, red, and green, indicate the Control, TC-0.6, and TC-3.0 groups of mice, respectively (mean ± S.E.M, n = 5/group). * indicates a significant difference from the Control group. (*P*<0.05, two-way ANOVA followed by Tukey's post hoc test).

The number of Arc-positive cells in the TC-0.6 group was found to be significantly decreased in ACC, and was increased in CeA and BLA, compared with the Control group and TC-3.0 group (Figs. 5A, 5B and 5C). Besides, a decreasing tendency was observed in PrL. On the other hand, no significant differences in the number of Arc-positive cells were observed in other 11 brain areas of the three groups of mice (Table 1). To study whether such an observation could be consistently obtained using another neuronal activation marker, we analyzed the levels of c-Fos expression in PrL, ACC, CeA and BLA. In the TC-0.6 group, the number of c-Fos-positive cells was found to be significantly decreased in the PrL and ACC, and was significantly increased in the CeA, compared with not only the Control but also TC-3.0 group (Fig. S5). That is, the numbers of Arc and c-Fos-positive cells in the TC-3.0 group were similar to that of the Control group. Thus, the signs of hypo-activation in the mPFC and hyper-activation in the amygdala in the TC-0.6 group represent a potential biological basis of the observed severe behavioral alterations, which supports the non-monotonic dose-response relationship observed in the behavioral test.

**Table 1:** Numbers of Arc-positive cells in 14 brain areas.

Area Symbol	Brain Area	Control	TC-0.6	TC-3.0
PrL	Prelimbic cortex	163.4±23.8	117.9±11.7	129.4±23.7
IL	Infralimbic cortex	151.2±19.2	141.5±7.2	142.6±17.1
Orb	orbital cortex	171.1±15.7	132.7±8.9	156.3±23.6
ACC	anterior cingulate cortex	179.4±16.7	90.5±23.7	147.2±35.1
M1	primary motor cortex	140.6±5.9	138.2±15.1	158.1±32.9
S1BF	primary somatosensory cortex barrel field	172.9±23.3	156.6±29.4	177.3±35.1
dlCPu	dorsal lateral caudate putamen	125.3±14.7	132.9±7.6	138.8±13.9
AcbC	nucleus accumbens core	182.3±22.0	174.2±24.1	190.1±27.6
RSGc	retrosplenial granular cortex	118.2±29.2	99.3±20.8	105.3±18.1
CA1	CA1 field of hippocampus	529.1±51.1	566.1±37.5	558.8±23.8
CA3	CA3 field of hippocampus	321.6±51.5	256.3±15.0	283.7±36.3
Ect	ectorhinal cortex	110.8±15.3	95.8±21.3	106.9±13.8
BLA	basolateral nucleus of the amygdala	62.5±14.7	113.5±22.3	82.7±7.3
CeA	central nucleus of the amygdala	88.9±25.3	182.2±6.7	141.4±17.6

Legend:

Data are shown as average ± S.E.M., n = 5/group. The values of PrL, ACC, BLA, and CeA are same as the data shown in Fig. 5C.

## Discussion

Using our recently developed behavioral task for group-housed mice, we investigated how a low dose of perinatal dioxin exposure affects executive function and social-emotional behavior in adulthood and analyzed immediate early gene products to determine the neurobiological basis of the observed behavioral alterations. We found the following four major findings in the TCDD-exposed mice: (i) impairment of two components of executive function, i.e., behavioral inflexibility and compulsive repetitive behavior, (ii) a dramatically lowered competitive dominance for limited sources of water reward after water deprivation, (iii) a remarkable association of the sign of altered mPFC and amygdala activities with behavioral abnormalities, and (iv) a robust, non-monotonic dose-response relationship in the behavioral and immunohistochemical assessments.

### Behavioral Inflexibility and Compulsive Repetitive Behavior

The first major finding was impairments in two components of the executive function, behavioral inflexibility and compulsive repetitive behavior, in the TCDD-exposed mice. Behavioral flexibility is the ability to adapt a series of habitual behaviors that are acquired in daily life to changes in the environment and it has been assessed using rule-shift learning task paradigms typified by reversal task [55], [56], [57]. We observed that the TCDD-exposed mice manifested behavioral inflexibility in the early and late stages of reversal learning.

In the early stage of reversal learning, which corresponds to the initial adaptation process, we found that all the groups of mice could facilitate their adaptation for the serial reversal task. They became “fast learners” by repetition of reversal tasks and were found to adapt in a considerably rapid manner to a reversed contingency as shown by the intra-session analysis in Rev.11, indicating the establishment of a “reversal learning-set” [58], [59]. However, in the first session of Rev. 2, the time when the Control group was found to markedly facilitate reversal learning compared to the first session of Rev.1, the TCDD-exposed groups were not able to shift their behavior to the reversed contingency as swiftly as the Control group, showing that TCDD-exposed mice had a delay in facilitation of reversal learning.

In the late stage of reversal learning, the TC-0.6 group was found to have a significantly lower degree of achievement in reversal learning than the Control group, indicating the occurrence of behavioral inflexibility in the maturing process of reversal learning. Because this result was comprehensively evaluated by analyzing all the data in Rev. 1- Rev. 11, we consider this trend consistent throughout the experiment. Furthermore, the difference in achievement between the Control and the TC-0.6 groups was more prominent in the sessions after Rev. 3, in which the reversal task was performed on a 4-session basis, compared with the sessions of Rev. 1 and Rev. 2, which were carried out on 7-session basis. In summary, it can be postulated that repetition of reversal learning during a short period made it difficult for the TC-0.6 group to sufficiently acquire reversal learning, and that the difference in degree of reversal learning attainment became more prominent between the Control and TC-0.6 group.

The TC-0.6 group was also found to show compulsive repetitive nose poking. In this study, the term compulsive is used to describe the perseverative character that manifested as repeated useless nose pokes in the corner that no longer yielded additional rewards. This phenotype was considered a sign of impairment in the inhibitory control of inappropriate behavior, a key component of executive function [60]. These two signs of impairment in executive function are related to the inability to inhibit or extinguish a behavior that no longer yields a desired outcome and have been associated with prefrontal dysfunction and observed in people with both neurodevelopmental and psychiatric disorders [61].

In contrast to our expectations, the actions of TCDD on executive function were much more prominent in the lower dose group (TC-0.6) than in the higher dose group (TC-3.0). The TC-0.6 group was found to have behavioral inflexibility and compulsive nose poking behavior consistently throughout the sessions, suggesting that the results are considered highly reproducible. It is intriguing that the TC-3.0 group did not show significant behavioral abnormalities compared to the Control, except for the learning performance in the first session of Rev. 2. As described in the Results section, the first session of Rev. 2 is unique in that it is the session in which mice relearned the previously acquired behavioral sequence for the first time. Thus, it is plausible to speculate that the TC-3.0 group had difficulty in applying the previously acquired knowledge to achieve a rapid behavioral shift. However, as shown in the inter-session analysis, such behavioral inflexibility in the TC-3.0 group might be masked by the training effect in the later sessions. The non-monotonicity of the action of TCDD is discussed later.

### Low Competitive Dominance in a Group-housed Environment

The second major finding of this study was low competitive dominance in the lower TCDD-exposed mice. In this study, the mice in the TC-0.6 group appeared hesitant to make an access to water reward at the beginning of the session. We first hypothesized that the TC-0.6 group had any physical abnormalities which resulted in their suppressed corner visit behavior. However, the TC-0.6 mice were not significantly different from the Control mice in terms of body size, motor function, daily water drinking behavior, and quick response to the start of the water-available period. In addition, the suppressed corner visit behavior was ameliorated by changing the social environment from a highly to less competitive condition by omitting the “dominant” group of mice. Thus, the low competitive dominance shown in the TC-0.6 group was confirmed to be attributable to social factors in the environment, and it can be referred to as social behavioral abnormality due to their possible altered social status. The present results suggest that perinatal exposure to TCDD may result in the development of a lifelong vulnerability to social-emotional challenges and induces a severe submissive or social-phobic temperament, as observed in human social anxiety disorder or ASD. In addition, the social defeat stress, to which the mice might have been subjected under such a low competitive dominance condition, could itself be a social risk factor or a causal contributor to the pathogenesis of mental or physical disorders, such as schizophrenia [62], [63]. Thus, it remains to be investigated whether or how abnormal competitive dominance is linked to the mental and physical health effects of TCDD exposure.

Only a few standardized behavioral methods are available to study the formation and maintenance of social status in rodents. For example, the tube test [64] and the urine-marking assay [65] has been applied to a pair of mice, whereas the visible burrow system [66] and the barber test [67] has been applied to more than three rodents in the group-housed condition. In comparison with these methods, our present method to assess competitive dominance has three unique features: (i) it can be applied to more than a dozen mice in a cage at once, (ii) it allows us to automatically monitor the establishment, maintenance, and transition of competitive dominance over a long period over days to months, (iii) the degree of competition can be easily manipulated by changing the number of animals per group and the composition of the experimental groups in a cage. These features will advance our understanding of the biological bases of the normal and pathological social behaviors observed in a diverse social structure.

### Signs of Altered Neuronal Activity in the mPFC and in the Amygdala

The third major finding of this study is the clear link between the behavioral alterations and the signs of hypo-activity in the mPFC and hyper-activity in the amygdala during the last session (Session 57). The mPFC and the amygdala are reciprocally connected to each other and regulate various cognitive and emotional processes, such as behavioral inhibition [68], the conditioning and extinction of fear [69], [70], [71], [72], and the response to stressful events [73], [74], [75], [76]. Perturbations in the functional connectivity or imbalances in the activity between the mPFC and the amygdala have been implicated in human mental disorders [77], [78]. Similar to the findings in human patients, rodent models of post-traumatic stress disorder and major depressive disorder-like behaviors induced by inescapable repeated tail-shocks [79], [80] and social defeat [81], [82] were found to show altered signs of neuronal activity in the mPFC and the amygdala [83]. This indicates that the imbalance of activity in these brain areas is strongly associated with the pathophysiological states of emotional dysfunction and that perinatal exposure to TCDD may contribute to the pathogenesis.

The TC-0.6 mice also shared a specific commonality in their pattern of neural activation with a recently reported mouse model of a submissive social state. Wang and associates showed that dominance rank in mice, as determined by the tube test, can be directly associated with the synaptic efficacy in the mPFC [47]. In their study, the level of excitatory synaptic inputs and the task-related c-Fos expression level in the mPFC were significantly reduced in subordinate mice, an observation which is consistent with our present result in the TC-0.6 mice that manifested as low competitive dominance in a group-housed environment. Interestingly, a recent study has also associated the establishment of ‘social hierarchy’ with amygdala activity in humans [45]. Thus, as indicated in the present study, it is possible that the altered activation of neurons in the amygdala, as well as in the mPFC, may be an additional determinant of abnormal social status in animals and humans.

### Non-monotonicity of Dioxin Actions

The fourth major finding of this study is the non-monotonic actions of TCDD on toxicological endpoints found in the lower-dose groups as alterations in the behavioral and neuronal activity indices (See also Fig. S4). It is a premise in pharmacology and toxicology that chemical exposure will exert a dose-dependent physiological response. This principle has been used as a dogma on which drug treatment and toxicity testing are based. However, in recent years, such a premise has been challenged by piles of evidence that shows the presence of non-monotonic modes of action of chemicals and hormones in a low-dose range and may elucidate the underlying mechanisms [84]. As the non-monotonic dose-response relationship found in this study was widely consistent among the indices of behavior and immunohistochemistry, the present results strongly suggest that TCDD is a chemical that can induce a non-monotonic dose-response effect on a systemic level, an observation that is consistent with previous studies on rat progeny [14], [19], [20]. To address mental disorders and related health problems as the endpoints of low-dose exposure of TCDD, further studies are required to clarify the non-monotonic association between a low dose of TCDD and its effects on the brain and behavior on both the molecular and systemic levels.

TCDD that was dosed in dams such as the present study can transfer to offspring brain tissue via *in utero* and lactational routes, from just after the exposure until weaning [50]. We also reported, in rats, that TCDD concentration of offspring brain decreased to a level close to the detection limit [15], indicating that the retained amounts of TCDD in adulthood, if any, is not likely to be large enough to affect the brain. Therefore, it is reasonable to suspect that a critical window of the brain development should be paid attention in terms of induction of the executive function deficits and low competitive dominance in adulthood. Further studies are needed to elucidate how TCDD, particularly at a low dose, disrupts neuronal development including neuronal migration, axonal elongation and synaptic formation.

### Advances in Higher Brain Function Behavior Analysis of Mice

In this study, we made important methodological advances in the behavioral analysis of higher brain function in mice. First, the present test was shown to be useful for assessing multiple aspects of cognitive behavior in mice, including a social-emotional aspect. In addition to the acquisition of place discrimination and behavioral sequence, behavioral flexibility, and reversal learning-set examined in the previous study [41], we showed here that the assessment of compulsive repetitive nose poking and competitive dominance for limited sources of water reward after water deprivation are useful behavioral hallmarks of cognitive abnormalities in mice. Second, the continuous monitoring of home cage cognitive behaviors over a period of weeks can be achieved using the present behavioral flexibility test or a competition task, which provides reliable evidence and consistent data for interpreting complex mouse behaviors. These tests will be useful to monitor the intervention effects of drugs, toxicants, or other experimental manipulations on mouse behavior during a long-term experiment.

## Supporting Information

Figure S1 **Validation of Arc antibody in Western blotting.** Western blotting was performed by the essentially same method as described previously (Kawashima et al., 2009). Mouse brain homogenates (10 µg protein/lane) were separated on standard SDS-polyacrylamide gels and transferred onto nylon membranes. The membranes were allowed to react with the anti-Arc pAb or the antigen-absorbed anti-Arc aAb, and then chemiluminescence was detected using ECL-Plus reagent (GE Healthcare, Buckinghamshire, UK). Cell lysates prepared from Arc-transfected HEK293T cells were used as a positive control. Arc immunoreactivity was detected as a 55 kDa band in Arc-overexpressing HEK293T cells and in the mouse brain by Western blotting with the rabbit anti-Arc antibody used for immunohistochemistry. The band disappeared when the antibody was pre-treated with an excess amount of recombinant Arc protein (right blot, antigen-absorption+).
(TIF)

Figure S2 **Photographs of mice in IntelliCage apparatus.** (A) Group-housed condition in IntelliCage. (B) Nose poking behavior in a corner chamber. An inset and a large picture each shows a mouse that is before and doing a nose poke.
(TIF)

Figure S3 **Basal activity in open field test and motor coordination tested by accelerating rota rod test.** No significant difference in the average travel distance and time spent in the center area (25 cm×25 cm) among groups was detected (A, B). No significant difference in latency to fall-down among groups was detected in accelerating rota rod test (C). Data are shown as average ± S.E.M., n = 8/group.
(TIF)

Figure S4 **Correlation coefficient matrix of 16 behavioral parameters described in Table S2 in Control (A), TC-0.6 (B) and TC-3.0 (C) mice.** Numbers in the table indicate the correlation coefficient between the corresponding two parameters. Bold face indicates a significant correlation (P<0.05, test of significance for Pearson product-moment correlation coefficient). Each table was made from data of all the animals or of each group. A total of 16 parameters were included. Numbers indicate a correlation coefficient between the corresponding two variables. Each cell is color coded according to the degree of correlation: negative correlation, no correlation, and positive correlation are shown in blue, white, and red, respectively. We found that the TC-0.6 group showed unique correlations among the following parameters. The variable of competitive dominance ([V 9]) was significantly correlated with both the number of impulsive nose pokes ([V 11]) and the behavioral flexibility scores ([V 13] and [V 14]). In contrast, such correlations were not observed in the Control and TC-3.0 groups. Thus, the behavior of the TC-0.6 group can be characterized by the strong correlations between competitive dominance, impulsivity and behavioral flexibility.
(TIF)

Figure S5 **Averaged time spent per visit (visit duration) during the first three 5-minute time frames after the task started throughout the behavioral flexibility test.** Bars, open, red, and green, indicate the Control, TC-0.6, and TC-3.0 groups of mice, respectively (mean ± S.E.M, n = 8/group).
(TIF)

Figure S6 **c-Fos-positive cells in each brain area as estimated by stereological analysis.** Bars, open, red, and green, indicate the Control, TC-0.6, and TC-3.0 groups of mice, respectively (mean ± S.E.M, n = 5/group). * indicates a significant difference from other two groups. (P<0.05, two-way ANOVA followed by Tukey's post hoc test).
(TIF)

Table S1 **The exploratory and spontaneous activity indices extracted from the acclimation phase 1 of IntelliCage test.** Data are shown as average ± S.E.M., n = 8/group.
(DOC)

Table S2 **Descriptions of observed behavioral variables extracted from IntelliCage test (prepared for Figure S4).**
(DOC)

## References

[1] Grandjean P, Landrigan PJ (2006) Developmental neurotoxicity of industrial chemicals. Lancet 368: 2167–2178. 17174709 10.1016/S0140-6736(06)69665-7

[2] Lidow MS, Song ZM (2001) Primates exposed to cocaine in utero display reduced density and number of cerebral cortical neurons. J Comp Neurol 435: 263–275. 11406810 10.1002/cne.1028

[3] Szpir M (2006) New thinking on neurodevelopment. Environ Health Perspect 114: A100–107. 16451834 10.1289/ehp.114-a100 PMC1367862

[4] Yang Y, Roussotte F, Kan E, Sulik KK, Mattson SN, et al (2011) Abnormal Cortical Thickness Alterations in Fetal Alcohol Spectrum Disorders and Their Relationships with Facial Dysmorphology. Cereb Cortex 10.1093/cercor/bhr193 PMC3328347 21799209

[5] Boyle CA, Decoufle P, Yeargin-Allsopp M (1994) Prevalence and health impact of developmental disabilities in US children. Pediatrics 93: 399–403. 7509480

[6] Fombonne E (2003) Epidemiological surveys of autism and other pervasive developmental disorders: an update. J Autism Dev Disord 33: 365–382. 12959416 10.1023/a:1025054610557

[7] Robison LM, Skaer TL, Sclar DA, Galin RS (2002) Is attention deficit hyperactivity disorder increasing among girls in the US? Trends in diagnosis and the prescribing of stimulants. CNS Drugs 16: 129–137. 11825103 10.2165/00023210-200216020-00005

[8] Patandin S, Lanting CI, Mulder PG, Boersma ER, Sauer PJ, et al (1999) Effects of environmental exposure to polychlorinated biphenyls and dioxins on cognitive abilities in Dutch children at 42 months of age. J Pediatr 134: 33–41. 9880446 10.1016/s0022-3476(99)70369-0

[9] Stewart PW, Lonky E, Reihman J, Pagano J, Gump BB, et al (2008) The relationship between prenatal PCB exposure and intelligence (IQ) in 9-year-old children. Environ Health Perspect 116: 1416–1422. 18941588 10.1289/ehp.11058 PMC2569105

[10] Schecter A, Gasiewicz TA (2003) Dioxins and health. New York, NY: Wiley-Interscience. xvi, 952 p.

[11] Guo YL, Lambert GH, Hsu CC (1995) Growth abnormalities in the population exposed in utero and early postnatally to polychlorinated biphenyls and dibenzofurans. Environ Health Perspect 103 Suppl 6: 117–122. 10.1289/ehp.95103s6117 PMC1518940 8549457

[12] Gray LE Jr, Kelce WR, Monosson E, Ostby JS, Birnbaum LS (1995) Exposure to TCDD during development permanently alters reproductive function in male Long Evans rats and hamsters: reduced ejaculated and epididymal sperm numbers and sex accessory gland weights in offspring with normal androgenic status. Toxicol Appl Pharmacol 131: 108–118. 7878665 10.1006/taap.1995.1052

[13] Hojo R, Stern S, Zareba G, Markowski VP, Cox C, et al (2002) Sexually dimorphic behavioral responses to prenatal dioxin exposure. Environ Health Perspect 110: 247–254. 11882475 10.1289/ehp.02110247 PMC1240764

[14] Ikeda M, Mitsui T, Setani K, Tamura M, Kakeyama M, et al (2005) In utero and lactational exposure to 2,3,7,8-tetrachlorodibenzo-p-dioxin in rats disrupts brain sexual differentiation. Toxicol Appl Pharmacol 205: 98–105. 15885269 10.1016/j.taap.2004.09.010

[15] Kakeyama M, Sone H, Miyabara Y, Tohyama C (2003) Perinatal exposure to 2,3,7,8-tetrachlorodibenzo-p-dioxin alters activity-dependent expression of BDNF mRNA in the neocortex and male rat sexual behavior in adulthood. Neurotoxicology 24: 207–217. 12606293 10.1016/S0161-813X(02)00214-0

[16] Mably TA, Moore RW, Goy RW, Peterson RE (1992) In utero and lactational exposure of male rats to 2,3,7,8-tetrachlorodibenzo-p-dioxin. 2. Effects on sexual behavior and the regulation of luteinizing hormone secretion in adulthood. Toxicol Appl Pharmacol 114: 108–117. 1585363 10.1016/0041-008x(92)90102-x

[17] Takeda T, Matsumoto Y, Koga T, Mutoh J, Nishimura Y, et al (2009) Maternal exposure to dioxin disrupts gonadotropin production in fetal rats and imprints defects in sexual behavior. J Pharmacol Exp Ther 329: 1091–1099. 19276399 10.1124/jpet.109.151282

[18] Haijima A, Endo T, Zhang Y, Miyazaki W, Kakeyama M, et al (2010) In utero and lactational exposure to low doses of chlorinated and brominated dioxins induces deficits in the fear memory of male mice. Neurotoxicology 31: 385–390. 20398696 10.1016/j.neuro.2010.04.004

[19] Hojo R, Kakeyama M, Kurokawa Y, Aoki Y, Yonemoto J, et al (2008) Learning behavior in rat offspring after in utero and lactational exposure to either TCDD or PCB126. Environ Health Prev Med 13: 169–180. 19568902 10.1007/s12199-008-0026-0 PMC2698261

[20] Markowski VP, Cox C, Preston R, Weiss B (2002) Impaired cued delayed alternation behavior in adult rat offspring following exposure to 2,3,7,8-tetrachlorodibenzo-p-dioxin on gestation day 15. Neurotoxicol Teratol 24: 209–218. 11943508 10.1016/s0892-0362(02)00186-1

[21] Mitsui T, Sugiyama N, Maeda S, Tohyama C, Arita J (2006) Perinatal exposure to 2,3,7,8-tetrachlorodibenzo-p-dioxin suppresses contextual fear conditioning-accompanied activation of cyclic AMP response element-binding protein in the hippocampal CA1 region of male rats. Neurosci Lett 398: 206–210. 16442728 10.1016/j.neulet.2005.12.087

[22] Powers BE, Lin TM, Vanka A, Peterson RE, Juraska JM, et al (2005) Tetrachlorodibenzo-p-dioxin exposure alters radial arm maze performance and hippocampal morphology in female AhR mice. Genes Brain Behav 4: 51–59. 15660668 10.1111/j.1601-183X.2004.00098.x

[23] Schantz SL, Seo BW, Moshtaghian J, Peterson RE, Moore RW (1996) Effects of gestational and lactational exposure to TCDD or coplanar PCBs on spatial learning. Neurotoxicol Teratol 18: 305–313. 8725643 10.1016/s0892-0362(96)90033-1

[24] Seo BW, Powers BE, Widholm JJ, Schantz SL (2000) Radial arm maze performance in rats following gestational and lactational exposure to 2,3,7,8-tetrachlorodibenzo-p-dioxin (TCDD). Neurotoxicol Teratol 22: 511–519. 10974589 10.1016/s0892-0362(00)00070-2

[25] Seo BW, Sparks AJ, Medora K, Amin S, Schantz SL (1999) Learning and memory in rats gestationally and lactationally exposed to 2,3,7,8-tetrachlorodibenzo-p-dioxin (TCDD). Neurotoxicol Teratol 21: 231–239. 10386826 10.1016/s0892-0362(98)00049-x

[26] Widholm JJ, Seo BW, Strupp BJ, Seegal RF, Schantz SL (2003) Effects of perinatal exposure to 2,3,7,8-tetrachlorodibenzo-p-dioxin on spatial and visual reversal learning in rats. Neurotoxicol Teratol 25: 459–471. 12798963 10.1016/s0892-0362(03)00014-x

[27] Kakeyama M, Sone H, Tohyama C (2001) Changes in expression of NMDA receptor subunit mRNA by perinatal exposure to dioxin. Neuroreport 12: 4009–4012. 11742229 10.1097/00001756-200112210-00031

[28] Mitsuhashi T, Yonemoto J, Sone H, Kosuge Y, Kosaki K, et al (2010) In utero exposure to dioxin causes neocortical dysgenesis through the actions of p27Kip1. Proc Natl Acad Sci U S A 107: 16331–16335. 20805476 10.1073/pnas.1002960107 PMC2941332

[29] Chapman DE, Schiller CM (1985) Dose-related effects of 2,3,7,8-tetrachlorodibenzo-p-dioxin (TCDD) in C57BL/6J and DBA/2J mice. Toxicol Appl Pharmacol 78: 147–157. 4035666 10.1016/0041-008x(85)90314-x

[30] Chen CH, Suckling J, Lennox BR, Ooi C, Bullmore ET (2011) A quantitative meta-analysis of fMRI studies in bipolar disorder. Bipolar Disord 13: 1–15. 10.1111/j.1399-5618.2011.00893.x 21320248

[31] Hill E (2004) Executive dysfunction in autism. Trends Cogn Sci 8: 26–32. 14697400 10.1016/j.tics.2003.11.003

[32] Hill E, Bird C (2006) Executive processes in Asperger syndrome: patterns of performance in a multiple case series. Neuropsychologia 44: 2822–2835. 16930637 10.1016/j.neuropsychologia.2006.06.007

[33] Kerns J, Nuechterlein K, Braver T, Barch D (2008) Executive functioning component mechanisms and schizophrenia. Biol Psychiatry 64: 26–33. 18549874 10.1016/j.biopsych.2008.04.027

[34] Kipp K (2005) A developmental perspective on the measurement of cognitive deficits in attention-deficit/hyperactivity disorder. Biol Psychiatry 57: 1256–1260. 15949996 10.1016/j.biopsych.2005.03.012

[35] Marazziti D, Consoli G, Picchetti M, Carlini M, Faravelli L (2010) Cognitive impairment in major depression. Eur J Pharmacol 626: 83–86. 19835870 10.1016/j.ejphar.2009.08.046

[36] Royall D, Lauterbach E, Cummings J, Reeve A, Rummans T, et al (2002) Executive control function: a review of its promise and challenges for clinical research. A report from the Committee on Research of the American Neuropsychiatric Association. J Neuropsychiatry Clin Neurosci 14: 377–405. 12426407 10.1176/jnp.14.4.377

[37] Chan R, Shum D, Toulopoulou T, Chen E (2008) Assessment of executive functions: review of instruments and identification of critical issues. Arch Clin Neuropsychol 23: 201–216. 18096360 10.1016/j.acn.2007.08.010

[38] Koechlin E, Summerfield C (2007) An information theoretical approach to prefrontal executive function. Trends Cogn Sci 11: 229–235. 17475536 10.1016/j.tics.2007.04.005

[39] Miller E (2000) The prefrontal cortex and cognitive control. Nat Rev Neurosci 1: 59–65. 11252769 10.1038/35036228

[40] Robbins T, Arnsten A (2009) The neuropsychopharmacology of fronto-executive function: monoaminergic modulation. Annu Rev Neurosci 32: 267–287. 19555290 10.1146/annurev.neuro.051508.135535 PMC2863127

[41] Endo T, Maekawa F, Voikar V, Haijima A, Uemura Y, et al (2011) Automated test of behavioral flexibility in mice using a behavioral sequencing task in IntelliCage. Behav Brain Res 221: 172–181. 21377499 10.1016/j.bbr.2011.02.037

[42] Adolphs R (2009) The social brain: neural basis of social knowledge. Annu Rev Psychol 60: 693–716. 18771388 10.1146/annurev.psych.60.110707.163514 PMC2588649

[43] Skuse D, Morris J, Lawrence K (2003) The amygdala and development of the social brain. Ann N Y Acad Sci 1008: 91–101. 14998875 10.1196/annals.1301.010

[44] Zink CF, Stein JL, Kempf L, Hakimi S, Meyer-Lindenberg A (2010) Vasopressin modulates medial prefrontal cortex-amygdala circuitry during emotion processing in humans. J Neurosci 30: 7017–7022. 20484643 10.1523/JNEUROSCI.4899-09.2010 PMC2880169

[45] Zink CF, Tong Y, Chen Q, Bassett DS, Stein JL, et al (2008) Know your place: neural processing of social hierarchy in humans. Neuron 58: 273–283. 18439411 10.1016/j.neuron.2008.01.025 PMC2430590

[46] Sapolsky RM (2005) The influence of social hierarchy on primate health. Science 308: 648–652. 15860617 10.1126/science.1106477

[47] Wang F, Zhu J, Zhu H, Zhang Q, Lin Z, et al (2011) Bidirectional control of social hierarchy by synaptic efficacy in medial prefrontal cortex. Science 334: 693–697. 21960531 10.1126/science.1209951

[48] WHO (1998) Executive Summary Report of “Assessment of the health risks of dioxins: re-evaluation of the Tolerable Daily Intake (TDI)”. 10.1080/713810655 10912238

[49] Kawashima T, Okuno H, Nonaka M, Adachi-Morishima A, Kyo N, et al (2009) Synaptic activity-responsive element in the Arc/Arg3.1 promoter essential for synapse-to-nucleus signaling in activated neurons. Proc Natl Acad Sci U S A 106: 316–321. 19116276 10.1073/pnas.0806518106 PMC2629236

[50] Kakeyama M, Tohyama C (2003) Developmental neurotoxicity of dioxin and its related compounds. Ind Health 41: 215–230. 12916752 10.2486/indhealth.41.215

[51] Galsworthy MJ, Amrein I, Kuptsov PA, Poletaeva II, Zinn P, et al (2005) A comparison of wild-caught wood mice and bank voles in the Intellicage: assessing exploration, daily activity patterns and place learning paradigms. Behav Brain Res 157: 211–217. 15639172 10.1016/j.bbr.2004.06.021

[52] Lu J, Zhang YH, Chou TC, Gaus SE, Elmquist JK, et al (2001) Contrasting effects of ibotenate lesions of the paraventricular nucleus and subparaventricular zone on sleep-wake cycle and temperature regulation. J Neurosci 21: 4864–4874. 11425913 10.1523/JNEUROSCI.21-13-04864.2001 PMC3508730

[53] Tse D, Takeuchi T, Kakeyama M, Kajii Y, Okuno H, et al (2011) Schema-dependent gene activation and memory encoding in neocortex. Science 333: 891–895. 21737703 10.1126/science.1205274

[54] Paxinos G, Franklin KBJ (2004) The mouse brain in stereotaxic coordinates. Amsterdam; Boston: Elsevier Academic Press. 1 v.

[55] Brigman JL, Graybeal C, Holmes A (2010) Predictably irrational: assaying cognitive inflexibility in mouse models of schizophrenia. Front Neurosci 4. 10.3389/neuro.01.013.2010 PMC2938983 20859447

[56] Chudasama Y, Robbins TW (2006) Functions of frontostriatal systems in cognition: comparative neuropsychopharmacological studies in rats, monkeys and humans. Biol Psychol 73: 19–38. 16546312 10.1016/j.biopsycho.2006.01.005

[57] Jones B, Mishkin M (1972) Limbic lesions and the problem of stimulus–reinforcement associations. Exp Neurol 36: 362–377. 4626489 10.1016/0014-4886(72)90030-1

[58] Schusterman RJ (1964) Successive Discrimination-Reversal Training and Multiple Discrimination Training in One-Trial Learning by Chimpanzees. J Comp Physiol Psychol 58: 153–156. 14197033 10.1037/h0044309

[59] van der Plasse G, Feenstra MG (2008) Serial reversal learning and acute tryptophan depletion. Behav Brain Res 186: 23–31. 17714800 10.1016/j.bbr.2007.07.017

[60] Spreen O, Strauss E (1998) A compendium of neuropsychological tests: administration, norms, and commentary. New York: Oxford University Press. xvi, 736 p. p.

[61] Lipszyc J, Schachar R (2010) Inhibitory control and psychopathology: a meta-analysis of studies using the stop signal task. J Int Neuropsychol Soc 16: 1064–1076. 20719043 10.1017/S1355617710000895

[62] McEwen BS, Tucker P (2011) Critical biological pathways for chronic psychosocial stress and research opportunities to advance the consideration of stress in chemical risk assessment. Am J Public Health 101 Suppl 1: S131–139. 22021312 10.2105/AJPH.2011.300270 PMC3222511

[63] Meyer-Lindenberg A, Tost H (2012) Neural mechanisms of social risk for psychiatric disorders. Nat Neurosci 15: 663–668. 22504349 10.1038/nn.3083

[64] Lindzey G, Winston H, Manosevitz M (1961) Social dominance in inbred mouse strains. Nature 191: 474–476. 13762409 10.1038/191474a0

[65] Desjardins C, Maruniak JA, Bronson FH (1973) Social rank in house mice: differentiation revealed by ultraviolet visualization of urinary marking patterns. Science 182: 939–941. 4745598 10.1126/science.182.4115.939

[66] Albeck DS, McKittrick CR, Blanchard DC, Blanchard RJ, Nikulina J, et al (1997) Chronic social stress alters levels of corticotropin-releasing factor and arginine vasopressin mRNA in rat brain. J Neurosci 17: 4895–4903. 9169547 10.1523/JNEUROSCI.17-12-04895.1997 PMC6573358

[67] Long SY (1972) Hair-nibbling and whisker-trimming as indicators of social hierarchy in mice. Anim Behav 20: 10–12. 4677163 10.1016/s0003-3472(72)80167-2

[68] Churchwell JC, Morris AM, Heurtelou NM, Kesner RP (2009) Interactions between the prefrontal cortex and amygdala during delay discounting and reversal. Behav Neurosci 123: 1185–1196. 20001103 10.1037/a0017734 PMC2902158

[69] Bishop SJ (2007) Neurocognitive mechanisms of anxiety: an integrative account. Trends Cogn Sci 11: 307–316. 17553730 10.1016/j.tics.2007.05.008

[70] Kim MJ, Loucks RA, Palmer AL, Brown AC, Solomon KM, et al (2011) The structural and functional connectivity of the amygdala: from normal emotion to pathological anxiety. Behav Brain Res 223: 403–410. 21536077 10.1016/j.bbr.2011.04.025 PMC3119771

[71] Ochsner KN, Gross JJ (2005) The cognitive control of emotion. Trends Cogn Sci 9: 242–249. 15866151 10.1016/j.tics.2005.03.010

[72] Quirk GJ, Beer JS (2006) Prefrontal involvement in the regulation of emotion: convergence of rat and human studies. Curr Opin Neurobiol 16: 723–727. 17084617 10.1016/j.conb.2006.07.004

[73] Etkin A, Egner T, Peraza DM, Kandel ER, Hirsch J (2006) Resolving emotional conflict: a role for the rostral anterior cingulate cortex in modulating activity in the amygdala. Neuron 51: 871–882. 16982430 10.1016/j.neuron.2006.07.029

[74] Pezawas L, Meyer-Lindenberg A, Drabant EM, Verchinski BA, Munoz KE, et al (2005) 5-HTTLPR polymorphism impacts human cingulate-amygdala interactions: a genetic susceptibility mechanism for depression. Nat Neurosci 8: 828–834. 15880108 10.1038/nn1463

[75] Radley JJ, Sawchenko PE (2011) A common substrate for prefrontal and hippocampal inhibition of the neuroendocrine stress response. J Neurosci 31: 9683–9695. 21715634 10.1523/JNEUROSCI.6040-10.2011 PMC3197245

[76] Veer IM, Oei NY, Spinhoven P, van Buchem MA, Elzinga BM, et al (2011) Endogenous cortisol is associated with functional connectivity between the amygdala and medial prefrontal cortex. Psychoneuroendocrinology 10.1016/j.psyneuen.2011.12.001 22204928

[77] Etkin A, Wager TD (2007) Functional neuroimaging of anxiety: a meta-analysis of emotional processing in PTSD, social anxiety disorder, and specific phobia. Am J Psychiatry 164: 1476–1488. 17898336 10.1176/appi.ajp.2007.07030504 PMC3318959

[78] Shin LM, Wright CI, Cannistraro PA, Wedig MM, McMullin K, et al (2005) A functional magnetic resonance imaging study of amygdala and medial prefrontal cortex responses to overtly presented fearful faces in posttraumatic stress disorder. Arch Gen Psychiatry 62: 273–281. 15753240 10.1001/archpsyc.62.3.273

[79] Foa EB, Zinbarg R, Rothbaum BO (1992) Uncontrollability and unpredictability in post-traumatic stress disorder: an animal model. Psychol Bull 112: 218–238. 1454893 10.1037/0033-2909.112.2.218

[80] Maier SF (1984) Learned helplessness and animal models of depression. Prog Neuropsychopharmacol Biol Psychiatry 8: 435–446. 6385140

[81] Blanchard DC, Spencer RL, Weiss SM, Blanchard RJ, McEwen B, et al (1995) Visible burrow system as a model of chronic social stress: behavioral and neuroendocrine correlates. Psychoneuroendocrinology 20: 117–134. 7899533 10.1016/0306-4530(94)e0045-b

[82] Krishnan V, Han MH, Graham DL, Berton O, Renthal W, et al (2007) Molecular adaptations underlying susceptibility and resistance to social defeat in brain reward regions. Cell 131: 391–404. 17956738 10.1016/j.cell.2007.09.018

[83] Hammack SE, Cooper MA, Lezak KR (2012) Overlapping neurobiology of learned helplessness and conditioned defeat: Implications for PTSD and mood disorders. Neuropharmacology 62: 565–575. 21396383 10.1016/j.neuropharm.2011.02.024 PMC3433056

[84] Vandenberg LN, Colborn T, Hayes TB, Heindel JJ, Jacobs DR Jr, et al (2012) Hormones and endocrine-disrupting chemicals: low-dose effects and nonmonotonic dose responses. Endocr Rev 33: 378–455. 22419778 10.1210/er.2011-1050 PMC3365860

