# Supplementary figures and images for: Executive Function Deficits and Social-Behavioral Abnormality in Mice Exposed to a Low Dose of Dioxin In Utero and via Lactation
Source: PLoS One. 2012 Dec 12;7(12):e50741. doi: 10.1371/journal.pone.0050741 (PMC3520971; doi:10.1371/journal.pone.0050741)

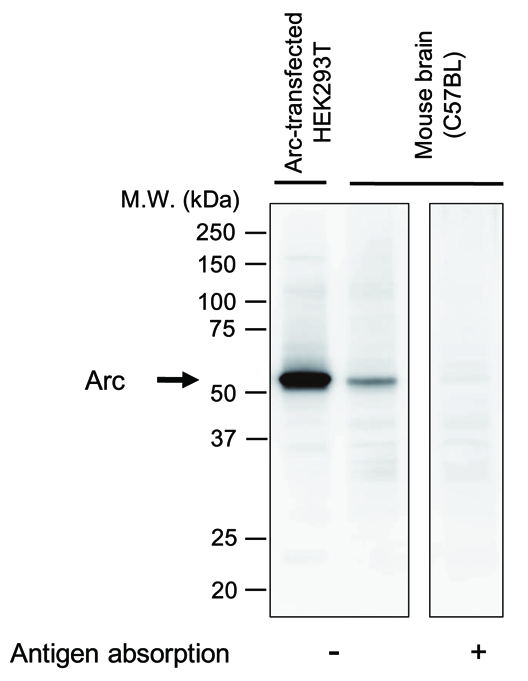

Supplement: Figure S1 — Validation of Arc antibody in Western blotting. Western blotting was performed by the essentially same method as described previously (Kawashima et al., 2009). Mouse brain homogenates (10 µg protein/lane) were separated on standard SDS-polyacrylamide gels and transferred onto nylon membranes. The membranes were allowed to react with the anti-Arc pAb or the antigen-absorbed anti-Arc aAb, and then chemiluminescence was detected using ECL-Plus reagent (GE Healthcare, Buckinghamshire, UK). Cell lysates prepared from Arc-transfected HEK293T cells were used as a positive control. Arc immunoreactivity was detected as a 55 kDa band in Arc-overexpressing HEK293T cells and in the mouse brain by Western blotting with the rabbit anti-Arc antibody used for immunohistochemistry. The band disappeared when the antibody was pre-treated with an excess amount of recombinant Arc protein (right blot, antigen-absorption+). (TIF) [file pone.0050741.s001.tif]

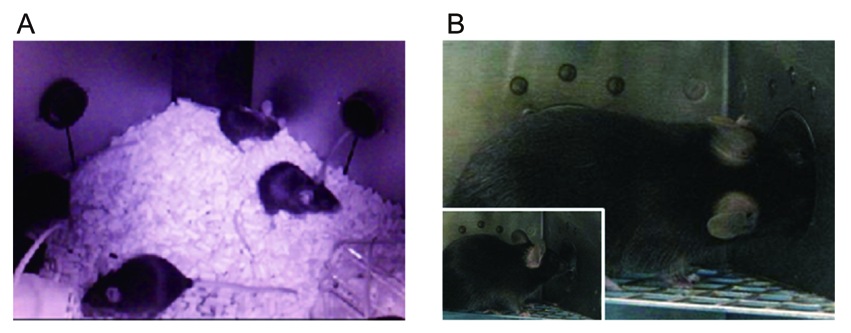

Supplement: Figure S2 — Photographs of mice in IntelliCage apparatus. (A) Group-housed condition in IntelliCage. (B) Nose poking behavior in a corner chamber. An inset and a large picture each shows a mouse that is before and doing a nose poke. (TIF) [file pone.0050741.s002.tif]

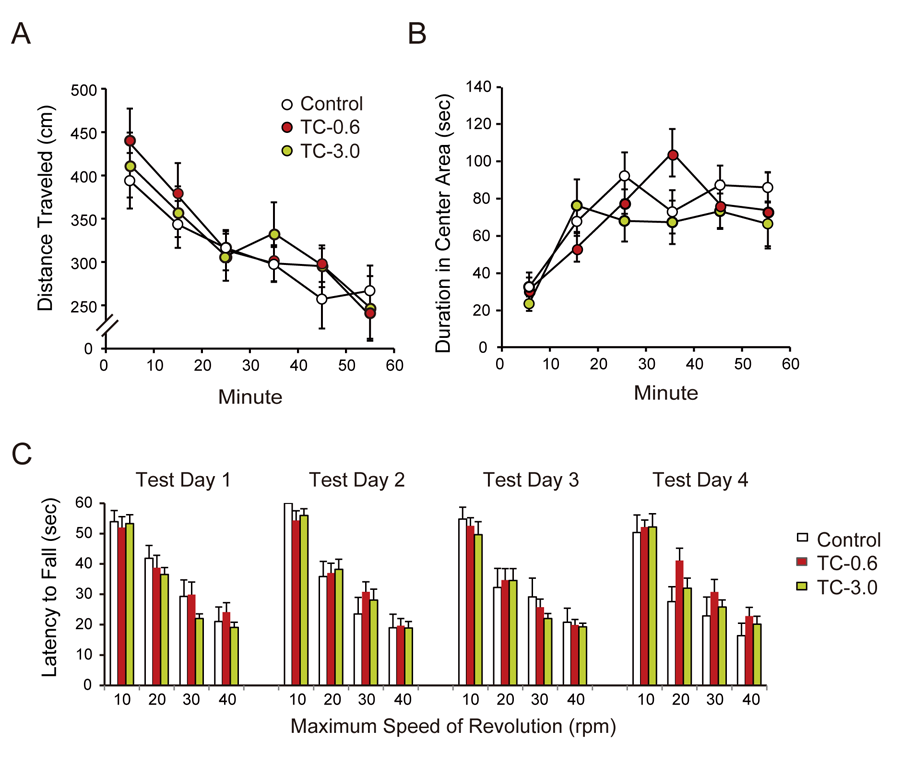

Supplement: Figure S3 — Basal activity in open field test and motor coordination tested by accelerating rota rod test. No significant difference in the average travel distance and time spent in the center area (25 cm×25 cm) among groups was detected (A, B). No significant difference in latency to fall-down among groups was detected in accelerating rota rod test (C). Data are shown as average ± S.E.M., n = 8/group. (TIF) [file pone.0050741.s003.tif]

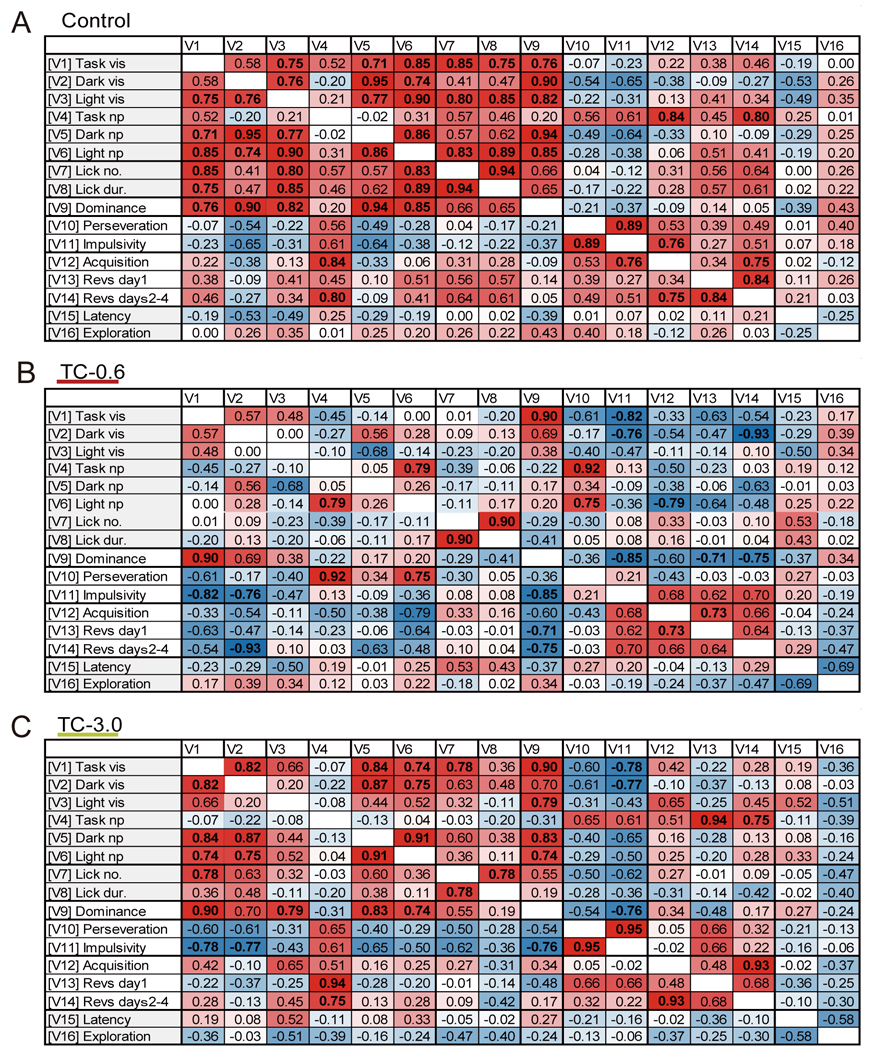

Supplement: Figure S4 — Correlation coefficient matrix of 16 behavioral parameters described in Table S2 in Control (A), TC-0.6 (B) and TC-3.0 (C) mice. Numbers in the table indicate the correlation coefficient between the corresponding two parameters. Bold face indicates a significant correlation (P<0.05, test of significance for Pearson product-moment correlation coefficient). Each table was made from data of all the animals or of each group. A total of 16 parameters were included. Numbers indicate a correlation coefficient between the corresponding two variables. Each cell is color coded according to the degree of correlation: negative correlation, no correlation, and positive correlation are shown in blue, white, and red, respectively. We found that the TC-0.6 group showed unique correlations among the following parameters. The variable of competitive dominance ([V 9]) was significantly correlated with both the number of impulsive nose pokes ([V 11]) and the behavioral flexibility scores ([V 13] and [V 14]). In contrast, such correlations were not observed in the Control and TC-3.0 groups. Thus, the behavior of the TC-0.6 group can be characterized by the strong correlations between competitive dominance, impulsivity and behavioral flexibility. (TIF) [file pone.0050741.s004.tif]

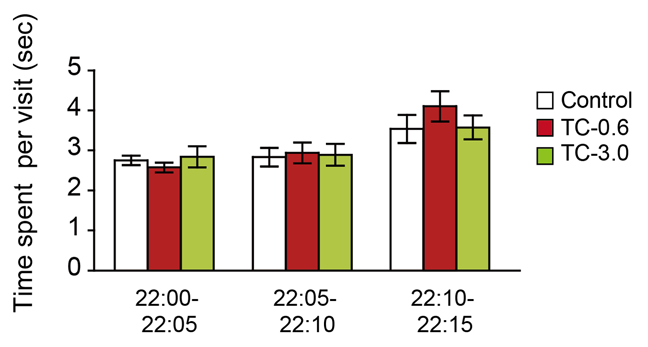

Supplement: Figure S5 — Averaged time spent per visit (visit duration) during the first three 5-minute time frames after the task started throughout the behavioral flexibility test. Bars, open, red, and green, indicate the Control, TC-0.6, and TC-3.0 groups of mice, respectively (mean ± S.E.M, n = 8/group). (TIF) [file pone.0050741.s005.tif]

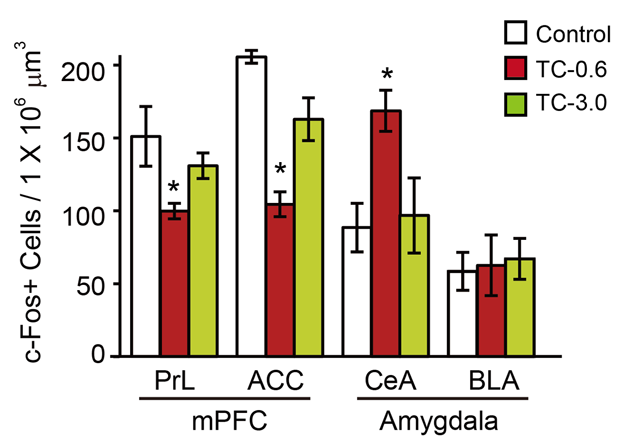

Supplement: Figure S6 — c-Fos-positive cells in each brain area as estimated by stereological analysis. Bars, open, red, and green, indicate the Control, TC-0.6, and TC-3.0 groups of mice, respectively (mean ± S.E.M, n = 5/group). * indicates a significant difference from other two groups. (P<0.05, two-way ANOVA followed by Tukey's post hoc test). (TIF) [file pone.0050741.s006.tif]
