# Supplementary material for: Executive Function Deficits and Social-Behavioral Abnormality in Mice Exposed to a Low Dose of Dioxin In Utero and via Lactation
Source: PLoS One. 2012 Dec 12;7(12):e50741. doi: 10.1371/journal.pone.0050741 (PMC3520971; doi:10.1371/journal.pone.0050741)
Supplement: Table S1 — The exploratory and spontaneous activity indices extracted from the acclimation phase 1 of IntelliCage test. Data are shown as average ± S.E.M., n = 8/group. (DOC) [file pone.0050741.s007.doc]

Table S1. The exploratory and spontaneous activity indices extracted from the acclimation phase 1 of IntelliCage test (data are shown as average ± S.E.M., n=8/group)

| Index | Control | TC-0.6 | TC-3.0 |
| --- | --- | --- | --- |
| Latency to the first corner visit (sec) | 23.5 ± 7.8 | 61.2 ± 28.9 | 55.9 ± 19.7 |
| Number of visits during the first 2 hrs | 16.0 ± 0.6 | 9.9 ± 3.5 | 6.1 ± 3.0 |
| 〃 4 hrs | 43.4 ± 19.5 | 24.8 ± 7.9 | 28.3 ± 8.8 |
| 〃 6 hrs | 78.6 ± 25.2 | 39.6 ± 17.9 | 59.7 ± 17.0 |
| 〃 8 hrs (including the first 2 hrs of the dark phase) | 131.6 ± 26.2 | 88.9 ± 24.7 | 92.1 ± 19.0 |
| Total number of visits / day | 192.6 ± 18.5 | 193.2 ± 17.1 | 177.9 ± 20.6 |
| 〃 during the light phase | 74.7 ± 11.5 | 70.4 ± 9.3 | 64.6 ± 9.9 |
| 〃 during the dark phase | 117.8 ± 13.3 | 122.7 ± 11.3 | 113.3 ± 15.8 |
| Circadian index (visits) | 100 ± 33.0 | 121.3 ± 24.6 | 116.7 ± 33.8 |
| Total number of lickings / day | 691.3 ± 84.7 | 529.3 ± 35.0 | 525.9 ± 62.6 |
| 〃 during the light phase | 289.5 ± 49.4 | 222.1 ± 26.4 | 201.4 ± 29.4 |
| 〃 during the dark phase | 477.9 ± 70.0 | 338 ± 32.4 | 329.8 ± 35.2 |
| Circadian index (lickings) | 100 ± 22.9 | 78.9 ± 32.8 | 96.9 ± 24.1 |
| % access to the most preferred corner | 30.8 ± 0.6 | 29.9 ± 28.9 | 31.2 ± 0.5 |
| 〃 the second preferred corner | 25.7 ± 0.2 | 27.0 ± 0.8 | 26.1 ± 0.5 |
| 〃 the third preferred corner | 23.5 ± 0.7 | 2.2 ± 0.6 | 23.2 ± 0.8 |
| 〃 the fourth preferred corner | 20.1 ± 0.9 | 2.3 ± 0.8 | 19.5 ± 0.7 |
| % nose poke to the preferred side | 60.0 ± 1.5 | 55.8 ± 1.6 | 57.2 ± 1.8 |
