# Supplementary material for: Executive Function Deficits and Social-Behavioral Abnormality in Mice Exposed to a Low Dose of Dioxin In Utero and via Lactation
Source: PLoS One. 2012 Dec 12;7(12):e50741. doi: 10.1371/journal.pone.0050741 (PMC3520971; doi:10.1371/journal.pone.0050741)
Supplement: Table S2 — Descriptions of observed behavioral variables extracted from IntelliCage test (prepared for Figure S4). (DOC) [file pone.0050741.s008.doc]

Table S2. Descriptions of observed behavioral variables extracted from IntelliCage test (Prepared for Figure S3)

| Variable ID | Variable Name | Description |
| --- | --- | --- |
| V1 | Visits in session | number of corner visits during the session period (22:00-1:00) |
| V2 | Visits in dark | number of corner visits during dark phase (20:00-8:00) except the session period |
| V3 | Visits in light | number of corner visits during light phase (8:00-20:00) |
| V4 | NPs in session | number of nose pokes during the session period |
| V5 | NPs in dark | number of nose pokes during dark phase except the session period |
| V6 | NPs in light | number of nose pokes during light phase |
| V7 | Lickings | number of corner visits during the session period |
| V8 | Licking duration | duration of licking during the session period |
| V9 | Dominance | number of corner visits during the first five minutes in each session (22:00-22:05) |
| V10 | Compulsive NP | number of nose pokes per visit to the rewarded corner (gate to the reward can be opened once per visit by an initial nose poke) |
| V11 | Impulsive NP | number of nose pokes per visit to the non-rewarded corner (gate to the reward cannot be opened) |
| V12 | Acquisition err. | averaged z-normalized scores of discrimination error rates in acquisition phase (Sessions 1 - 7) |
| V13 | Early stage err. | averaged z-normalized scores of discrimination error rates in the first sessions of each Rev. |
| V14 | Late stage err. | averaged z-normalized scores of discrimination error rates in the second to fourth sessions of each Rev. |
| V15 | Latency | latency to the first corner visit after mice were introduced to IntelliCage for the first time |
| V16 | Exploration | number of corner visits during the first two hours after mice were introduced to IntelliCage for the first time |
